# Tribocorrosion and Abrasive Wear Test of 22MnCrB5 Hot-Formed Steel

**DOI:** 10.3390/ma15113892

**Published:** 2022-05-30

**Authors:** Dariusz Ulbrich, Arkadiusz Stachowiak, Jakub Kowalczyk, Daniel Wieczorek, Waldemar Matysiak

**Affiliations:** 1 Faculty of Civil and Transport Engineering, Poznan University of Technology, Marii Sklodowskiej-Curie 5 sq., 60-965 Poznan, Poland; arkadiusz.stachowiak@put.poznan.pl (A.S.); jakub.kowalczyk@put.poznan.pl (J.K.); daniel.wieczorek@put.poznan.pl (D.W.); 2 Faculty of Mechanical Engineering, Poznan University of Technology, Marii Sklodowskiej-Curie 5 sq., 60-965 Poznan, Poland; waldemar.matysiak@put.poznan.pl

**Keywords:** tribocorrosion, abrasive wear, hot-forming process, boron steel

## Abstract

The article presents the results of research on abrasive and tribocorrosion wear of boron steel. This type of steel is used in the automotive and agricultural industries for the production of tools working in soil. The main goal of the article is the evaluation of tribocorrosion and abrasive wear for hot-formed 22MnCrB5 steel and a comparison of the obtained results with test results for steel in a cold-formed state. The spinning bowl method to determine the wear of samples working in the abrasive mass was used. Furthermore, a stand developed based on the ball-on-plate system allows to determine the wear during the interaction of friction and corrosion. After the hot-forming process, 22MnCrB5 steel was three times more resistant for the abrasive wear than steel without this treatment. The average wear intensity for 22MnCrB5 untreated steel was 0.00046 g per km, while for 22MnCrB5 hot-formed steel it was 0.00014 g per km. The tribocorrosion tests show that the wear trace of hot-formed 22MnCrB5 steel was about 7.03 µm, and for cold-formed 22MnCrB5 steel a 12.11 µm trace was noticed. The hot-forming method allows to obtain the desired shape of the machine element and improves the anti-wear and anti-corrosion properties for boron steel.

## 1. Introduction

Manufacturers of machines and vehicles seek to extend their failure-free operation through scheduled maintenance, which reduces the wear of individual elements. Excessive wear of cooperating parts results in disturbances in proper operation and the necessity to perform repairs. This situation generates machine downtime and additional costs. The wear of machine elements cannot be stopped; therefore, it is aimed at limiting it with various methods. For many years, both manufacturers of machines as well as scientists have conducted research on reducing the parts’ wear [1,2,3]. As well as the wear of machine elements during their operation, corrosive processes take place which have a destructive effect on the condition of the elements [4,5].

An example of a complex wear process is tribocorrosion [6,7]. In this process, the material loss of friction nodes in machines as a result of simultaneous friction and corrosion effects occurs. Generally, tribocorrosion is the synergy of friction and corrosion that determines the loss of material. Most often, such a mechanism of the synergy effect is observed for materials covered with a layer of passive oxides in a corrosive environment [8,9,10,11]. This protective layer is removed due to the frictional effects. Intensive electrochemical processes are initiated on the exposed surface of the material. On the other hand, in the case of materials that do not show the ability of passivation, the factors causing greater corrosion wear in the friction area may be the movement of the electrolyte or a change in the stress state in the surface layers [12,13].

The increase in resistance to only mechanical wear (abrasion) is usually achieved by increasing the hardness of the material. Unfortunately, most of the classic technological treatments that produce such an effect (heat treatment, plastic working) reduce the corrosion resistance [14]. The selection of the optimal material solution to minimize tribocorrosive wear is difficult [15,16,17].

One of the methods of improving the durability and strength of machine elements is to use a different material with better mechanical properties [18]. Nevertheless, this type of change is not always possible, especially in the case of the influence on the steel elements of several factors. An example of this problem is the wear of elements working in the ground (soil), where both abrasive and corrosive factors act together. In this case, additional modification of the material or its surface layer can be applied, which increase the resistance to wear and corrosion [19,20].

The wear of steel elements of agricultural machinery in soil mass is defined as a phenomenon of shearing, grooving, scratching, or cutting processes, causing the destruction of the surface layer of the material [21]. In addition, in abrasive wear, the properties of the abrasive mass, such as grain size composition, moisture, and compactness of the soil, should be considered [22,23,24]. Napiórkowski [25] presented a comprehensive study to understand the relationship between the physicochemical properties of soil, agrotechnical extortion, or properties of materials, and the wear process of the element of an agricultural machine. The obtained dependencies allowed for changes in the process of the design and selection of steel for the ploughshare, depending on various soil conditions. Similar studies were carried out by Natsis et al. [26], in which a strong correlation between soil type and share wear for different values of share hardness was established. Other factors influencing abrasive wear include the working time and the depth of the element’s immersion in the abrasive mass, as well as the hardness of the material [27,28,29].

22MnB5 steel has been used for several years in the construction of car bodies, especially for elements that require high strength. The main advantages of this material solution (steel + forming technology) are:

- High abrasion resistance of boron steel.
- Large possibilities of forming complex shapes (greater than in the case of cold-forming).

Therefore, as part of various tests, the properties of this steel are verified, especially after heat treatment processes [30]. Merklein [31] found that the temperature increase during the hot-stamping process for 22MnB5 steel results in a significant decrease of the flow stress values and the slope of the initial strain hardening. Escosa et al. [32] conducted a wear test of 22MnB5 steel using the pin-on-disc test. The research result showed that AlSi reduces the friction coefficient during the test. The electrochemical behavior of 22MnB5 steel coated with hot-dip AlSi before and after the hot-stamping process was investigated by Couto et al. [32]. These studies confirmed that the hot-stamping process changes the coating morphology, and consequently, the electrochemical behavior. A significant change in the anodic/cathodic coupling of the coating/steel due to iron enrichment in the coating layer was observed. Other studies assess the effect of the applied coating on steel corrosion [33,34]. Allely et al. [35] tested anticorrosion mechanisms of aluminized steel for hot stamping. The main conclusion from the test is that the AlSi coating can be applied for the 22MnB6 substrate to reduce the corrosion potential. Additionally, Park et al. [36] showed that hot-forming can provide better corrosion resistance to steel than cold-forming.

There are various methods of improving abrasive resistance, such as pad welding [37,38], hardening [39], laser treatment [40], or application of coatings with the required technological properties [41,42]. Other methods of increasing the resistance to abrasive wear include the use of cemented carbides [43] and oxide ceramics [44]. Considering the above technological possibilities, the authors proposed the hot-forming process as a method that allows for the modification of the structure and the surface layer, which may increase the corrosion resistance and wear resistance in relation to the basic material.

This article compares hot- and cold-formed 22MnCrB5 steel in terms of wear resistance under the following conditions:

- Intense abrasion in the abrasive mass—the influence of the shaping technology on the material properties (especially hardness) determining the abrasion resistance was analyzed.
- Tribocorrosion—combined action of friction and corrosive environment, where the influence of the shaping technology on the complex properties (hardness, corrosion resistance) was analyzed.

The tests were performed for samples cut from ready-made factory products. The subject of the analysis was the wear resistance under the conditions mentioned above, but not the material properties.

Boron steels are used in the construction of elements of agricultural and heavy machinery working in the ground (soil). Therefore, it is important to evaluate the influence of the hot-forming process on the resistance of tribocorrosion and abrasive wear of steel. The hot-forming process is used not only to change the shape of the elements, but also to change the internal structure, which can have a positive effect on wear and corrosion resistance. The tests were carried out in laboratory conditions on prepared stands that allowed to obtain the same conditions (humidity, pH, grain size) during the research.

The research was divided in two main stages. One of them included the tribocorrosion test, and the second one focused only on the abrasive wear test. The main goal of the article was achieved by performing the following tasks:

- Determination of material loss for tested samples of 22MnCrB5 (after the hot-forming process) under tribocorrosion conditions on a laboratory stand.
- Determination of material loss for tested samples of 22MnCrB5 (after the hot-forming process) under the abrasive wear test in a spinning bowl unit.
- Comparison of sample wear in the tribocorrosion test and abrasive wear test without the influence of corrosive factors, with results for 22MnCrB5 cold-formed steel.

The above tests allow to determine how the hot-forming process affects the wear resistance and corrosion resistance of boron steels. It is important from the point of view of the application of these steels, especially in the agricultural industry. Limiting the process of steel wear as well as corrosion through the hot-forming operation will allow for longer periods of use of agricultural tools, which is extremely important during field work.

## 2. Materials

**Figure 1:**
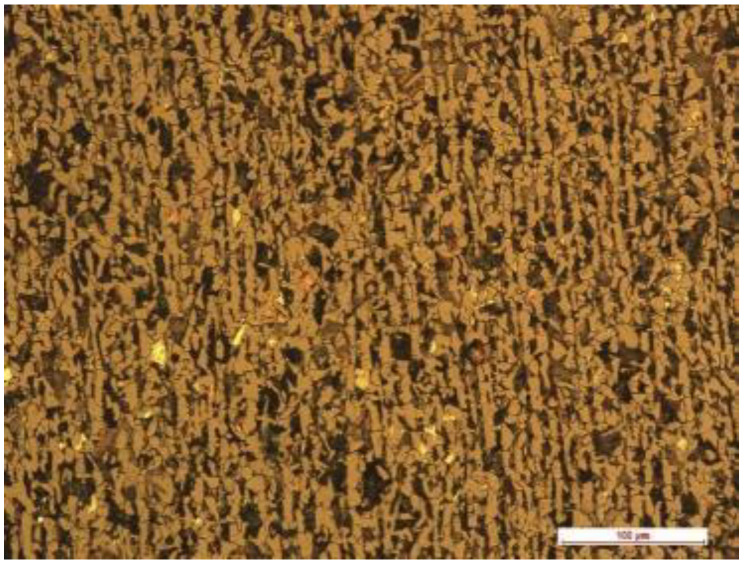
Microstructure of the tested 22MnCrB5 steel in the cold-formed state.

The resistance to tribocorrosion and abrasive wear was assessed for hot-formed 22MnCrB5 steel and compared to untreated boron steel. Materials used in the research contain boron. The mechanical properties of the material are presented in Table 1. Table 2 includes the chemical composition of the tested steel. The microstructure of the steel is presented in Figure 1, which includes a view of the 22MnCrB5 steel before the hot-forming process (cold-formed state). There is a hard pearlite phase embedded in ferrite. This is one of the boron-alloyed quenched and tempered steels. Steel is characterized by good formability in the hot-rolled state and high strength after the heat treatment. Low boron additions to the composition of the steel ensure high hardenability, better mechanical properties, and weight savings of up to 50% compared to conventional steel used in the automotive and agriculture industry. Boron is also added to the steel to increase the hardenability as it retards the heterogeneous nucleation of ferrite at austenite grain boundaries. In addition, in the quenching process, shrinkage occurs. Therefore, parts are resistant to the aggressive conditions, including adhesive wear, abrasion, thermal stresses, and fatigue.

The hot-forming method is gaining importance due to the possibility of changing properties inside the material, in accordance with technical requirements in terms of strength [45,46,47]. It allows to obtain a shape similar to the final product, as well as to adjust the microstructural properties in one production step [48]. To obtain specific properties of the final part in the form of a burr, it is necessary to use parameters such as time and temperature in a controlled manner. Thanks to this, it is possible to maintain appropriate structural changes in the material during the heating, annealing, and cooling process. This approach allows gradation of hardness and strength along the thickness or length of the part. The scope of the transition zone and the achievement of precisely located hard and soft areas with different zone properties was possible mainly due to the ability to control the exact range of temperature during the heating of the blank. The main advantages of the hot-forming process are the excellent shape accuracy of the burr, as well as the ability to produce ultra-high-strength parts without spring-back. It is due to the conversion of austenite to martensite during the pressing operation. In the literature, there are test results that present the hot-forming technology of new materials [49,50] as well as those that improve the entire process [51].

The hot-forming process consists of various stages: austenitization, blank transfer, hot-forming, and cutting. The first step is to heat the blank to 900–950 °C for a few minutes. At this temperature, the steels are very ductile and can easily be formed into complex shapes. The heating time depends on the thickness of the blanks (for our process and thickness of the steel, the heating time was around 25 s). Then, the operation of transferring the hot blank is performed. It is important to control the temperature of the blank so that it does not fall below 780 °C, as bainite and/or ferrite begin to form. During the stamping process, the hot blank is placed by the robotic arm in a tool—a die—which is cooled with water at ambient temperature. The tools are cooled down for approximately 15 s on the hydraulic press. The part is then taken from the tool at a temperature of around 80 °C to guarantee a good geometry after the last air-quench. The last stage is cutting the product to its final dimensions. The development of laser cutters, especially 3D, resulted in the use of these machines for cutting moldings made with the hot-forming technology.

**Figure 2:**
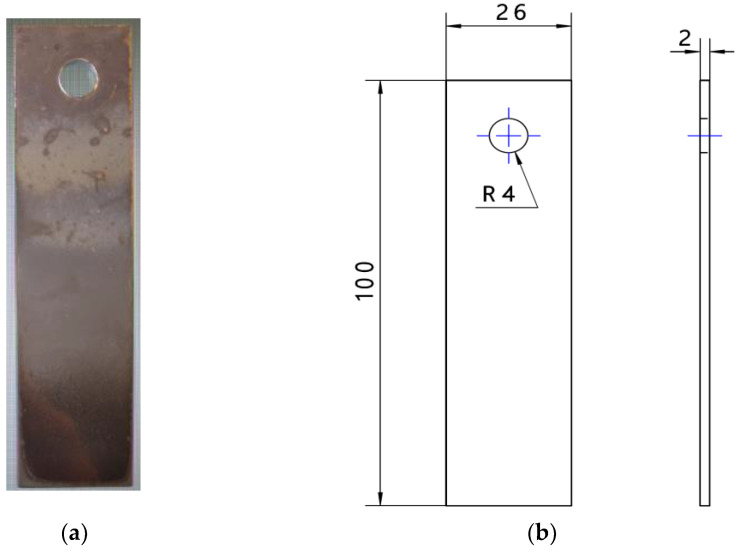
View (**a**) and dimensions (in mm) (**b**) of the samples used during the abrasive wear test.

Six identical samples of the tested steel, in accordance with the dimensions shown in Figure 2 (for the abrasive wear test), were prepared. The samples of 22MnCrB5 steel were cut from the finished machine element, which was manufactured in the hot-forming process. For untreated steel (cold-formed state), samples from a sheet of metal were cut. To evaluate the tribocorrosion of the tested steel, samples with a diameter of 10 mm and a thickness of 2 mm were prepared.

## 3. Research Methods

### 3.1. Tribocorrosion Test

**Figure 3:**
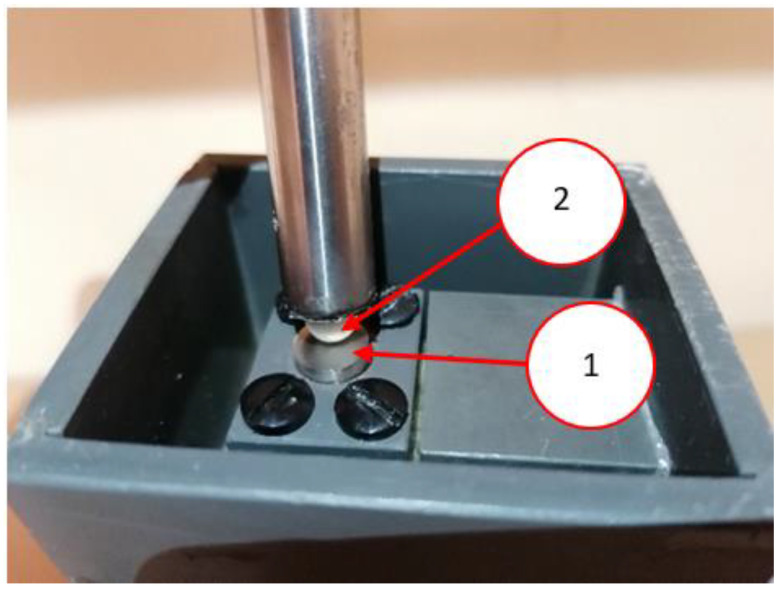
View of the sample during the tribocorrosion test: (**1**)—sample, (**2**)—Al_2_O_3_ ball.

Tribocorrosion tests were performed for a ball-on-plate model node using the stand (Figure 3) presented in the previous publication [52]. A sample made of the tested material had the shape of a cylinder with a diameter of 10 mm. Using a special holder, the sample was mounted inside a chamber filled with a corrosive environment (3.5% NaCl was used in the tests). The ball was moving on the surface of the sample in a reciprocating motion. The stroke length (distance between the extreme positions of the ball) was about 6 mm. In the tests, Al_2_O_3_ balls with a diameter of 7.0 mm and a load of about 9.6 N were used. Spheres with Al_2_O_3_ (as counter-samples) were used in the tests. The hardness of the balls was approximately 1800 HV. The balls were made in the G25 accuracy class. The contact force and ball diameter were selected according to the hardness of the tested materials [53].

Three research tests (each one lasted about 1 h) were performed for each of the tested materials. During this test, the ball made about 14,400 displacements (movement frequency 2 Hz). The duration of the test and the frequency of ball movement were selected to achieve measurable sample wear (above 2 µm). The wear of the sample was determined after the end of the tests based on profilometric measurements of the wear trace. The maximum depth of the wear trace (determined in the middle of the friction path) was assumed as the measure of wear. Measurements were performed using a Carl Zeiss ME-10 (Carl Zeiss AG, Jena, Germany) profilometer. The device made it possible to estimate the depth with an accuracy of about 0.01 µm.

Electrochemical measurements (determination of polarization curves) in a three-electrode system were carried out. The ATLAS 9833 potentiostat was used for this purpose. The reference electrode was the calomel electrode (SCE). The role of the auxiliary electrode was played by a platinum mesh. The area of the sample, except for the wear place, was covered with a tape made of non-conductive material. The essence of the tribocorrosion process is the occurrence of the synergy of friction and corrosion (ΔZ) according to the relationship:

(1) $$Z_{T}=Z_{M}+Z_{K}+\Delta Z$$

where Z_T_ is material loss under tribocorrosion conditions, Z_M_ is material loss caused only by mechanical effects, and Z_K_ is material loss caused only by corrosive effects.

To estimate the value of the component ΔZ, tests under the conditions of only mechanical impact were carried out. The cooperation of the ball–sample friction association took place in a corrosive environment, but with cathodic polarization eliminating the corrosive processes. The material loss determined in these conditions corresponds to the component Z_M_. The component Z_K_ was analytically calculated using the Faraday equation.

### 3.2. Abrasive Wear Test

**Figure 4:**
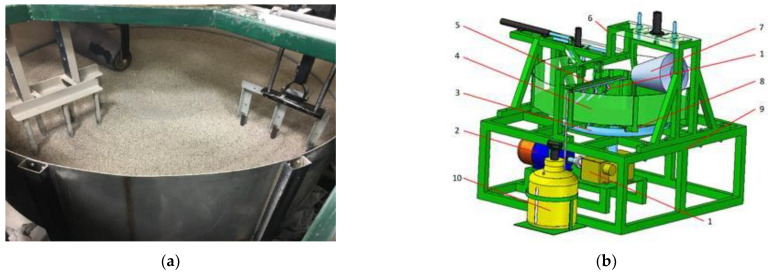
Spinning bowl unit: (**a**) view of the stand, (**b**) model of the stand: 1—transmission shaft, 2—engine, 3—travel rail, 4—bowl, 5—sample holder, 6—compacting roller frame, 7—compacting roller, 8—frame, 9—main frame, 10—water tank.

**Figure 5:**
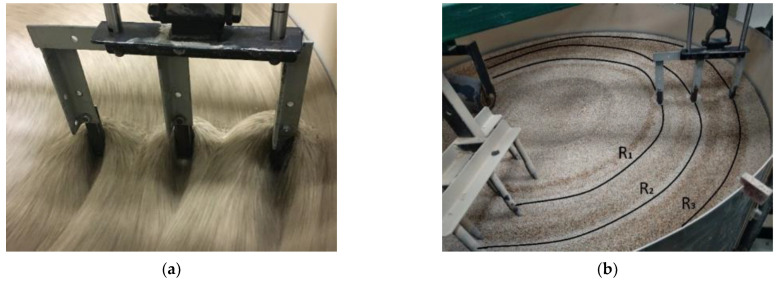
Distribution of samples on the stand: (**a**) view, (**b**) radius R_1_, R_2_, and R_3_, along which the sample moves on the stand.

The abrasive wear test on the laboratory stand, by the “spinning bowl unit” method, was performed. The view of the test stand is shown in Figure 4. The tests were carried out in sand with a grain diameter of 0.2–0.8 mm. The humidity level of the abrasive mass was also controlled and equaled around 1.5%. The rotational speed of the bowl unit allowed to obtain the working conditions in the field, which equaled an average speed of 6–7 km/h. The total distance covered by one sample during the test was 300 km. The weight of the samples was measured before starting the tests and after every 100 km of the distance test in the abrasive environment. After each measurement, the samples were replaced on the stand. It was caused by the distribution of samples on the holder (Figure 5), and samples covered different distances (radius R_1_, R_2_, and R_3_).

Samples’ weight measurements were each time preceded by cleaning in an ultrasonic bath to remove impurities. Then, the samples were dried at the temperature of 80 °C for 20 min. The samples prepared in this way were weighed on the device with the accuracy of ±0.001 g. The samples were placed in the same direction each time on the scale. Based on the test results, the weight loss of the samples in accordance with Equation (2) was determined:

(2) $$Z_{\mathrm{pw}}=m_{w}-m_{i}\;\left[g\right]$$

where *m_w_* is the initial sample mass before the test, in g, and *m_i_* is the sample mass after the friction test, in g.

Based on the above equation and data, the intensity of mass wear was calculated (3):

(3) $$I_{pw}=\frac{Z_{\mathrm{pw}}}{S}\;\left[\frac{g}{\mathrm{km}}\right]$$

where *S* is the friction distance, in km.

Additionally, the topography (surface roughness profile) for the samples tested in the abrasive wear process was measured. The T8000 contact profilograph and HommelMap Expert software by Hommelwerke were used to study the topography of the surface. The measuring tip TKL100/17 was used with a cone-shaped diamond needle with an angle of 90° and a tip radius of 2 μm. The probe travel speed was 0.15 mm/s. The dimensions of the recorded area were 1.5 × 1.5 mm. It consisted of 301 tracks separated by 0.005 mm. After registration, the surface was leveled, and then roughness was filtered using a cut-off wavelength of 0.25 mm. As a result of filtration, the outer surface fragments of 0.125 mm on each side were cut-off (the analyzed area was reduced to 1.25 × 1.25 mm).

## 4. Results of Research

### 4.1. Tribocorrosion Test Result

**Figure 6:**
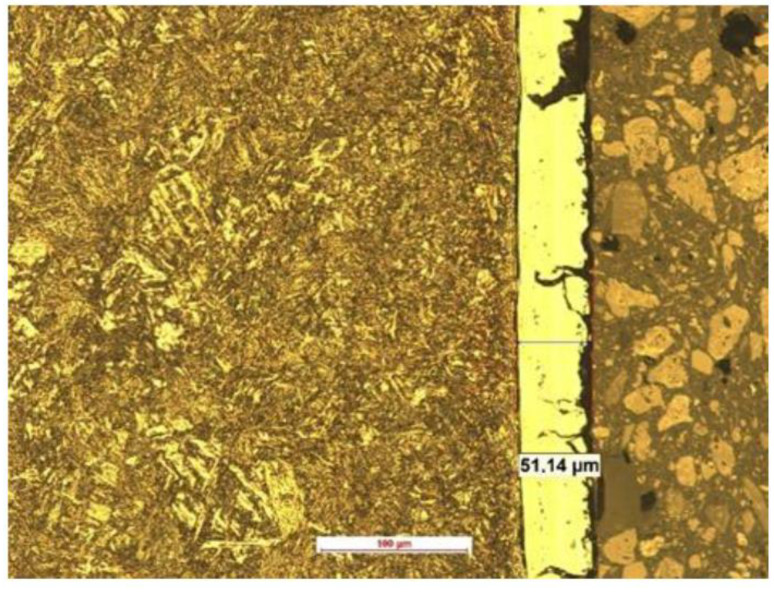
Microstructure of the tested 22MnCrB5 steel after the hot-forming process with the AlSi layer.

Figure 6 shows the martensitic structure, which was created in the hot-forming process. Additionally, it shows the AlSi layer with around 50 µm thickness that was applied to the steel surface to prevent oxidation during the hot-forming process.

**Figure 7:**
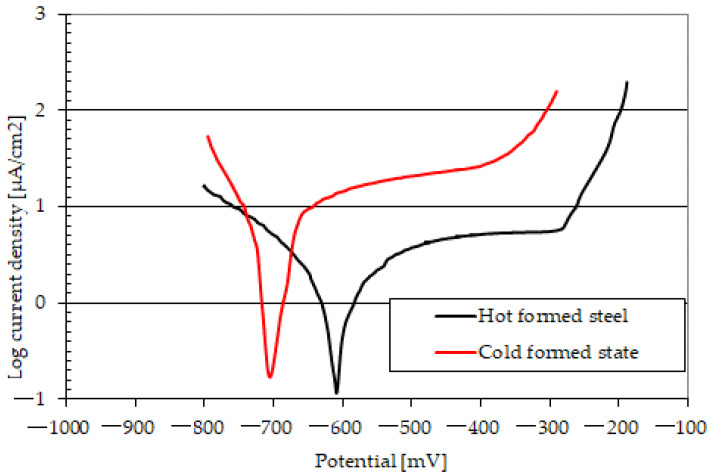
Polarization curves of tested materials in 3.5% NaCl (5 mV/s).

Before the tribocorrosion tests, the polarization curves of the tested materials were determined. The obtained results are shown in Figure 7. Three tests were performed for each material. To quantify the resistance of the tested steels to the corrosive action of 3.5% NaCl, the corrosion potential and the corrosion current density were determined. The Tafel method and the AtlasLab software were used for this purpose. The results are presented in Table 3. The obtained results have shown that the hot-forming process improves the material’s resistance to corrosion with 3.5% NaCl. The corrosion current density for 22MnCrB5 steel is several times lower for the same type of steel but in the cold-formed state.

**Figure 8:**
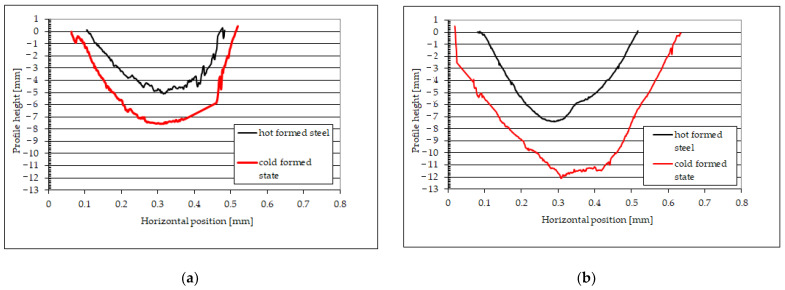
Examples of wear patterns: (**a**) only mechanical wear, (**b**) tribocorrosion.

Table 4 presents the material loss of the tested steels for the tribocorrosion process (Z_T_) and only mechanical wear (Z_M_). Only mechanical wear was determined under the conditions of cathodic polarization, using the polarization potential of 200 mV lower than the corrosion potential. For the comparative analysis, it was assumed that the main measure of material loss was the depth of the wear pattern. Figure 8 shows exemplary wear patterns. Table 5 contains the wear volume in the tribocorrosion test for a 6 mm track length.

During tribocorrosion research, the wear of 22MnCrB5 steel after the hot-forming process was about 7.03 µm, and for steel in the cold-formed state, about 12.11 µm. Under the conditions of only mechanical wear, the wear trace depth for the hot-formed 22MnCrB5 steel was approximately 5.01 µm, while for the untreated steel it was approximately 7.91 µm.

Based on the test results, the synergy effect of friction and corrosion (ΔZ) was also calculated. According to the information provided in Section 3.1, the material loss caused only by the corrosive effects (Z_K_) was estimated using the Faraday equation. The calculations were performed for the corrosion current density (Table 3) and the test duration (1 h). The depth of the removed material layer was approximately 0.003 µm for 22MnCrB5 hot-formed steel and 0.016 µm for steel in the cold-formed state. Due to the very small values of the component (Z_K_), the synergy effect of friction and corrosion was estimated as the difference (ΔZ = Z_T_ − Z_M_).

### 4.2. Abrasive Wear Test Result

**Figure 9:**
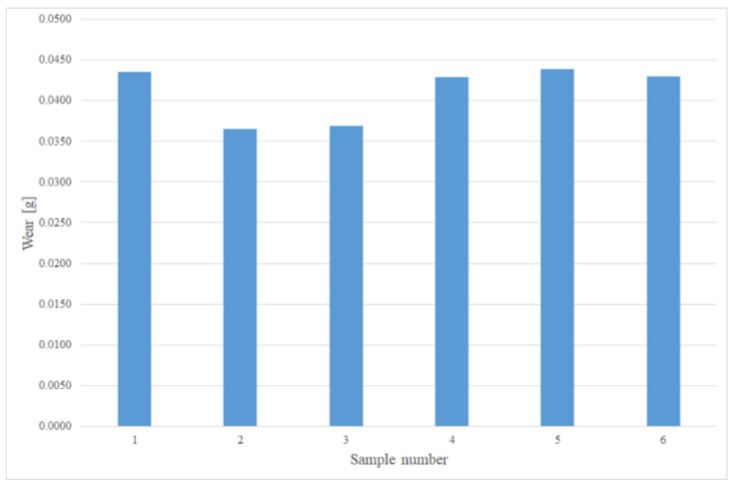
Wear of all samples, in g, for 22MnCrB5 steel after the hot-forming process.

**Figure 10:**
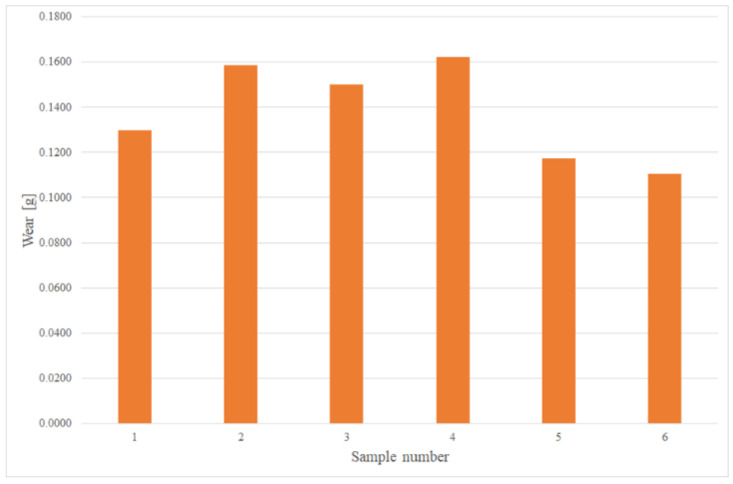
Wear of all samples, in g, for cold-formed 22MnCrB5 steel.

**Figure 11:**
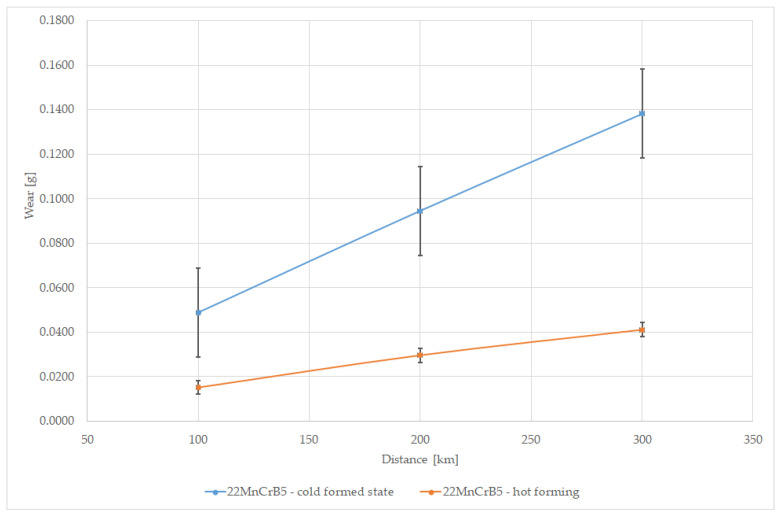
Comparison of the results of the average wear of samples depending on the distance in the abrasive mass on the spinning bowl unit.

The test results in the form of weight loss (wear) in grams for individual samples are shown in Figure 9 and Figure 10. Figure 11 presents a comparison of the average wear of the samples depending on the distance affected by the abrasive mass. The average wear intensity for cold-formed steel was 0.00046 g/km, while for the 22MnCrB5 steel sheet after the hot-forming process it was 0.00014 g/km. This means there was a three times greater resistance to the abrasive wear of steel after the hot-forming process. The results of the wear of samples are presented in grams because the sand pressure occurred from the front of the sample on the surface, regardless of the material from which it was made.

**Figure 12:**
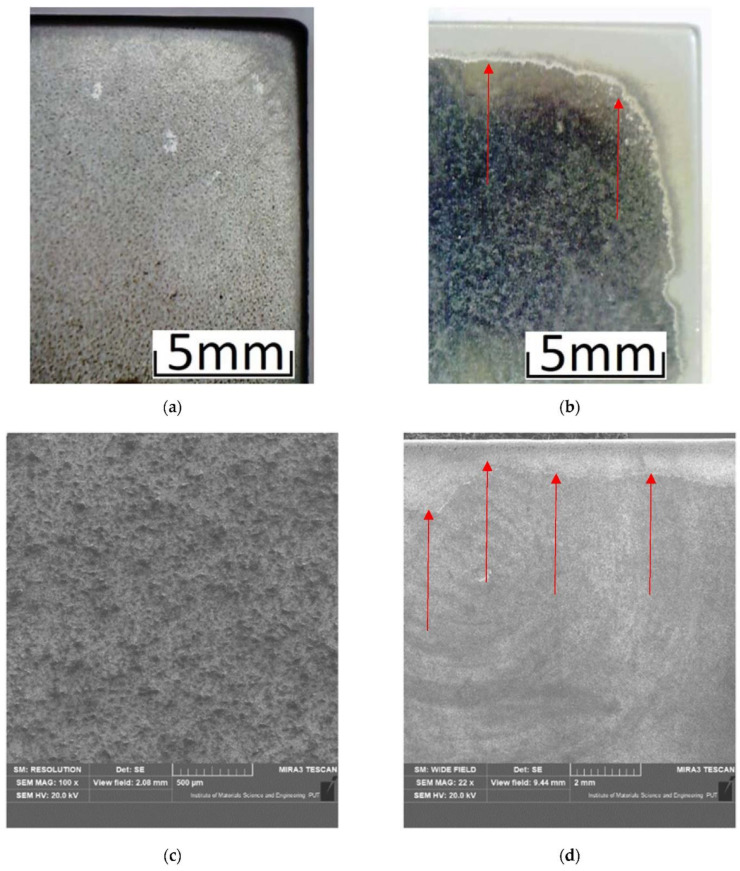
View of sample surface wear: (**a**,**c**) 22MnCrB5 in the cold-formed state, (**b**,**d**) 22MnCrB5 after the hot-forming process.

In addition to the weight loss test, the surface of the sample was verified to determine the wear model and the dominant damage mechanism of the surface layer. A selected view of the samples after the test in the abrasive mass is shown in Figure 12.

## 5. Discussion

The results of wear tests indicate that 22MnCrB5 hot-formed steel is characterized by a greater resistance to tribocorrosion (Table 4 and Table 5). In the case of this steel, a significantly smaller material loss was found under the conditions of simultaneous frictional and corrosive effects. The main reason for this is a significant increase in the hardness of the material obtained as a result of the hot-forming process. The higher hardness of hot-formed steel resulted in shallower wear marks (than for 22MnCrB5 steel in the cold-formed state) after tests under only mechanical impacts.

In the case of both tested steels, a clear synergy effect of friction and corrosion was found in the tribocorrosion process. Most likely, this effect was caused by the influence of the frictional interactions in the ball-on-plate sliding association on the corrosion processes. The synergy effect (ΔZ) was smaller for the 22MnCrB5 hot-formed steel, around 28%. The lower effects of the interaction of friction and corrosion resulted from the greater resistance of the hot-formed 22MnCrB5 steel to the corrosive action of 3.5% NaCl (Figure 6). It is worth emphasizing that in the analyzed case, the hot-forming process improved the hardness and corrosion resistance of the material. Consequently, it may be suitable for steels used in the construction of friction junctions operated in tribocorrosion conditions.

**Figure 13:**
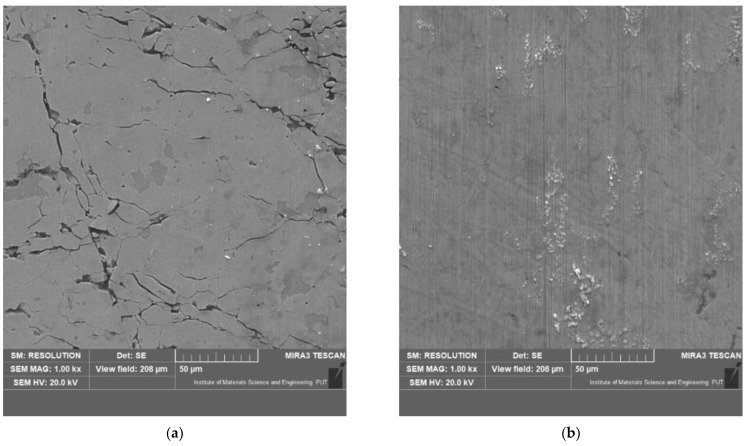

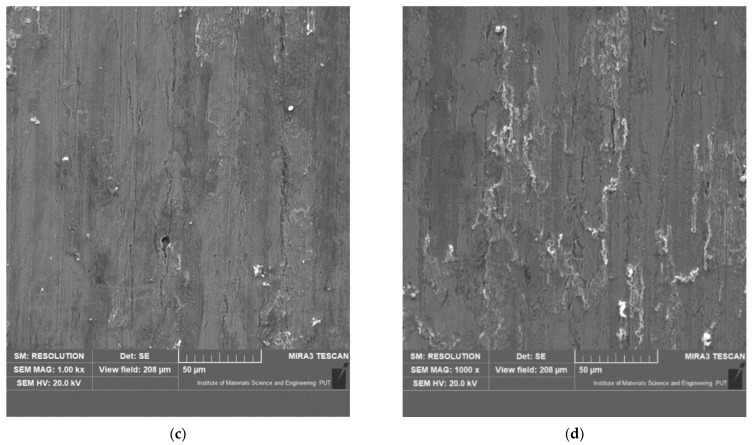
View of the surface after the wear tests: (**a**) 22MnCrB5 hot-formed steel—tribocorrosion test, (**b**) 22MnCrB5 hot-formed steel—mechanical wear test, (**c**) 22MnCrB5 cold-formed state—tribocorrosion test, (**d**) 22MnCrB5 cold-formed state—mechanical wear test.

Based on the microscopic observations of the surface of the wear trace, an attempt was made to identify the elementary mechanisms causing removal of the material. Sample views of the surface after tests are presented in Figure 13. In the case of only mechanical interactions, micro-cutting dominates for both samples. This is evidenced by parallel grooves in the direction of the ball sliding (counter samples). These traces were clearer and deeper for 22MnCrB5 steel in the cold-formed state (lower hardness of the sample). After tribocorrosion tests, traces of micro-cutting were less visible. This may be the result of the removal of the surface layers of the material by corrosive interactions. In the case of 22MnCrB5 hot-formed steel, numerous cracks are visible. This material is characterized by high hardness and may be prone to local cracking under conditions of high unit pressures. Such conditions prevail in a ball-on-plate association. The presence of the electrolyte and its corrosive effect favor the propagation of microcracks in the subsurface layers of the material.

**Figure 14:**
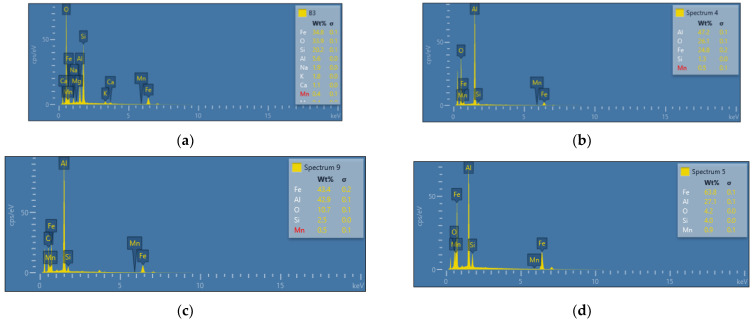
View of the chemical composition of the surface for 22MnCrB5 steel: (**a**) for cold-formed steel, and (**b**–**d**) for hot-formed steel. (**a**,**b**) The base material, (**c**) after the corrosion condition, and (**d**) after the tribocorrosion condition.

An additional layer of AlSi, which was applied to the surface of the sheet before the hot-forming process, had a significant influence on corrosion. This is a standard operation that primarily prevents oxidation of the sheet during the hot-stamping process. Therefore, Figure 14 shows the chemical composition of the surface layer before and after the test, depending on the treatment (cold-formed state and hot-formed state). In the case of the cold-formed state sample, the minimum Al values were observed. For samples after the hot-forming process, the content of this element was many times higher due to the additional coating. The influence of the tribocorrosion phenomenon causes the partial abrasion of this layer and the decrease of the Al content by about half in relation to the original value.

**Figure 15:**
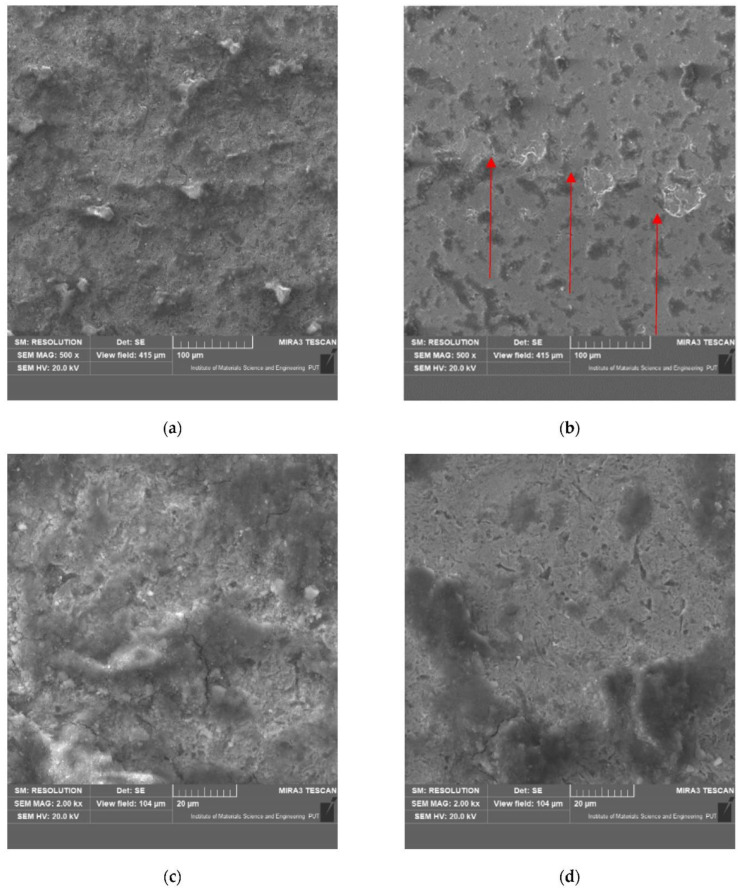
View of the surface after the wear tests: (**a**,**c**) 22MnCrB5 steel in cold-formed state, (**b**,**d**) 22MnCrB5 hot-formed steel.

Based on the wear tests of the samples in the abrasive mass on the spinning bowl unit, it was found that for the 22MnCrB5 steel (after the hot-forming process), over three times lower wear was determined than for the 22MnCrB5 cold-formed state steel. This fact can be due to the presence of martensite in the material structure and the additional AlSi layer. It was noticed that the wear intensity for 22MnCrB5 untreated steel was constant, while for 22MnCrB5 hot-formed steel, a slight decrease was observed. Tests were carried out over a distance of 300 km. The wear measurements were conducted every 100 km of the test. For 22MnCrB5 steel in the cold-formed state, it was found that the average wear of the samples was 0.00049 g/km for the first 100 km, and for the next 100 and 200 km, 0.00046 and 0.00044 g/km, respectively. For 22MnCrB5 steel (after the hot-forming process), the wear was 0.00015, 0.00014, and 0.00011 g/km, for each 100 km of the test in the spinning bowl unit. The average wear intensity for 22MnCrB5 untreated steel was 0.00046 g per km, while for 22MnCrB5 hot-formed steel it was 0.00014 g per km. Based on the view of the surface structure after the wear tests (Figure 15) in the abrasive mass, it was found that the main mechanism destroying the structure of the material was the crushing of particles from the surface layer. In the case of samples after the hot-forming process, damages of the AlSi layer were additionally noticed (Figure 11b,d and Figure 13b—the area of damage to the layer is marked with arrows). Moreover, for 22MnCrB5 cold-formed state steel, cracks in the surface layer of the material were visible in several places. A smaller number of cracks were also noticed for the 22MnCrB5 hot-formed steel, which occurred in the area where the AlSi coating was removed. However, in the case of the two tested samples, there were no observed particles in the surface layer, which was shown in [54] for boron steel.

**Figure 16:**
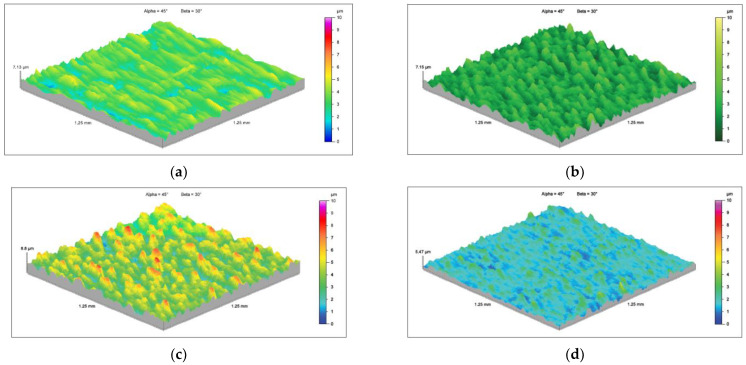
Roughness profile topography: (**a**) 22MnCrB5 cold-formed steel before the test, (**b**) 22MnCrB5 cold-formed steel after the test, (**c**) 22MnCrB5 hot-formed steel before the test, (**d**) 22MnCrB5 hot-formed steel after the test.

**Figure 17:**
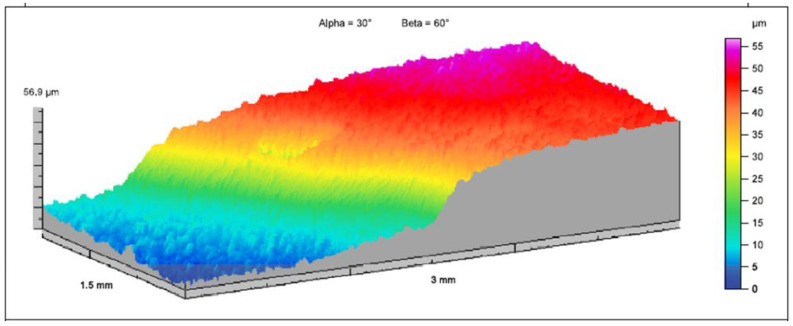
Roughness profile topography for the 22MnCrB5 hot-formed sample after the test with the decrease of the surface profile.

In addition, in the case of testing the wear resistance of materials in the abrasive mass, the surface roughness profile (surface topography) was verified both before and after the test using the spinning bowl method (Figure 16). For the roughness profile study, the surface was analyzed in the most worn area before the fault, and near the hole in the least worn area. Based on the obtained results, it was found that there were local increases in the roughness profile. This may be caused by impacts of the abrasive particles, which locally damaged the surface and crushed the material (this was also confirmed by cracks on the surface of the samples). This was especially visible for 22MnCrB5 cold-formed steel. In the case of steel after the hot-forming process, a greater smoothing of the surface of the samples was visible. Moreover, detachment of the AlSi coating was found. The wear of this coating is additionally illustrated in Figure 17. The additional coating allowed for the reduction of material wear.

Additional treatment in the form of the hot-forming process allowed to improve the anti-wear and anti-corrosion properties. Similar results of increasing the wear resistance were obtained by the authors of [55], who reduced the wear of boron steel (30MnCrB4) used for rotavator blades via cryogenic treatment. The main reason why the hot-formed samples have better corrosion and tribocorrosion resistance than cold-formed samples is the change in the microstructure of the steel. The pearlitic structure, which is the basic structure of 22MnCrB5 steel, was changed into a martensitic structure during the hot-forming process. Accordingly, the hardness of the samples changed. Additionally, to avoid oxidation during the hot-forming process, an AlSi layer was applied. As the test results show, this coating can additionally protect the material against corrosive agents.

## 6. Conclusions

Based on the performed research, the following conclusions can be made:

- In the case of both tested materials (in the cold-formed state and the hot-forming process), a clear synergy effect of friction and corrosion was identified in the tribocorrosion process. This effect was most likely caused by the influence of frictional interactions on the course of electrochemical phenomena on the material surface.
- The test results indicated that 22MnCrB5 hot-formed steel obtained significantly greater resistance to tribocorrosion. For this material, a smaller material loss was found after tribocorrosion tests, as well as a smaller share of the friction–corrosion synergy effect in total wear.
- The hot-forming process significantly improved the anti-wear properties of boron steel due to the change in the internal structure (mechanical properties) and an additional AlSi coating applied on the surface of the sample.
- The performed laboratory tests showed that the use of the hot-forming technology for boron steel significantly reduced the abrasive wear and limited the corrosion steel of the process. This may result in an increase in the operation time of agricultural and machinery components working in soil.

A disadvantage (limitation) related to the use of the technology of shaping machine elements by plastic deformation may be the differentiation of the structure, and consequently, the properties of the material on the surface. Unfortunately, such a state favors the development of corrosion (the presence of corrosive micro-cells). Therefore, based on the performed tests and the analysis of their results, within a certain range of conditions affecting the tested steels, the analyzed material solution and the hot-forming process can be used.

The directions of further research should include the comparison of wear and tribo-corrosion of steel subjected to the hot-formed process with steel that has undergone various heat treatments. This will allow to optimize the production of selected machine parts, especially in terms of wear and corrosion resistance, which may have a significant impact on the lifetime of a selected machine element.

## Figures and Tables

**Table 1:** Mechanical properties of the tested materials.

Properties	22MnCrB5
Hardness	165–235 HV, after hot-forming, 400–520 HV
Tensile strength (MPa)	400–570, after hot-forming, 1300–1650
Yield strength (MPa)	200
Elongation A_80_ (%)	15

**Table 2:** Chemical composition.

Material	C	Si	P	S	Mn	Al	Cr	Ti	B
22MnCrB5	0.19–0.25	0.4	0.025	0.015	1.10–1.40	0.02–0.06	0.15–0.25	0.02–0.05	0.0008–0.005

**Table 3:** Corrosion potential (Ecorr) and corrosion current density (icorr).

	22MnCrB5—Cold-Formed State	22MnCrB5 after Hot-Forming
Ecorr (mV) (SCE)	−710 ± 20	−620 ± 20
icorr (µA/cm^2^)	13 ± 1	2.3 ± 0.3

**Table 4:** Experimental research results.

Material	Material Loss in the Tribocorrosion (Z_T_)	Mechanical Wear (Z_M_)	(ΔZ = Z_T_ − Z_M_)	ΔZ/Z_T_
	(µm)	(µm)	(µm)	(%)
22MnCrB5 in cold-formed state	12.1 ± 0.2	7.9 ± 0.2	4.20	35
22MnCrB5 after hot-forming	7.0 ± 0.2	5.0 ± 0.2	2.02	28

**Table 5:** Calculation of wear volume.

Material	Material Loss in the Tribocorrosion (Z_T_)	Mechanical Wear (Z_M_)
	(mm^3^)	(mm^3^)
22MnCrB5 in cold-formed state	0.028 ± 0.001	0.015 ± 0.001
22MnCrB5 after hot-forming	0.013 ± 0.001	0.008 ± 0.001

## Data Availability

Not applicable.
